# Changes in performance and bio-mathematical model performance predictions during 45 days of sleep restriction in a simulated space mission

**DOI:** 10.1038/s41598-020-71929-4

**Published:** 2020-09-24

**Authors:** Erin E. Flynn-Evans, Crystal Kirkley, Millennia Young, Nicholas Bathurst, Kevin Gregory, Verena Vogelpohl, Albert End, Steven Hillenius, Yvonne Pecena, Jessica J. Marquez

**Affiliations:** 1 Fatigue Countermeasures Laboratory N262-4, Human Systems Integration Division, NASA Ames Research Center, Moffett Field, CA 94035 USA; 2 Fatigue Countermeasures Laboratory, Human Systems Integration Division, San José State University Research Foundation, Moffett Field, CA 94035 USA; 3 Biomedical Research and Environmental Sciences Division, Human Health and Performance Directorate, NASA Johnson Space Center, Houston, TX USA; 4 Department of Aviation and Space Psychology, German Aerospace Center (DLR), Hamburg, Germany; 5 Human Computer Interaction Group, Human Systems Integration Division, NASA Ames Research Center, Moffett Field, CA 94035 USA

**Keywords:** Human behaviour, Computational models

## Abstract

Lunar habitation and exploration of space beyond low-Earth orbit will require small crews to live in isolation and confinement while maintaining a high level of performance with limited support from mission control. Astronauts only achieve approximately 6 h of sleep per night, but few studies have linked sleep deficiency in space to performance impairment. We studied crewmembers over 45 days during a simulated space mission that included 5 h of sleep opportunity on weekdays and 8 h of sleep on weekends to characterize changes in performance on the psychomotor vigilance task (PVT) and subjective fatigue ratings. We further evaluated how well bio-mathematical models designed to predict performance changes due to sleep loss compared to objective performance. We studied 20 individuals during five missions and found that objective performance, but not subjective fatigue, declined from the beginning to the end of the mission. We found that bio-mathematical models were able to predict average changes across the mission but were less sensitive at predicting individual-level performance. Our findings suggest that sleep should be prioritized in lunar crews to minimize the potential for performance errors. Bio-mathematical models may be useful for aiding crews in schedule design but not for individual-level fitness-for-duty decisions.

## Introduction

Lunar habitation and exploration of space beyond low-Earth orbit poses many challenges. Lunar crews are likely to be comprised of small groups of individuals living in confined spaces for more than a month at a time, and mission support personnel may not be consistently available to assist crewmembers due to communication delays (https://www.nasa.gov/what-is-artemis). Prior spaceflight missions have established that crewmembers achieve less than the recommended number of hours of sleep per night on Earth^1^; however, few studies have been conducted to determine how the reduced sleep duration observed in space may relate to performance changes among rigorously selected individuals who may be intrinsically motivated to perform at a high level. Furthermore, it is unclear whether bio-mathematical models that are used to design work schedules on Earth would provide performance predictions consistent with actual performance during a spaceflight mission. If such models can accurately predict performance changes among crew during spaceflight, they could be used to allow crew to self-schedule activities around periods of cognitive vulnerability, potentially minimizing the reliance of crewmembers on mission control oversight.

Several studies have shown that astronauts achieve approximately 6 h of sleep per night while in space, but are able to sleep longer when on Earth^2–6^, suggesting that crewmembers accumulate a sleep deficit while in space. The few studies evaluating cognitive performance changes during spaceflight are mixed, with six studies including 23 crewmembers finding that inflight performance is reduced relative to on Earth^3,7–10^, and three studies including six crewmembers that did not find changes in performance during spaceflight missions^11–13^. These studies are limited by low sample size, few data collection points during a mission, and varying mission durations and schedules, making it difficult to interpret whether sleep loss could relate to the changes in performance observed in some studies.

Astronauts are a self-selected group of highly motivated individuals. Several studies have demonstrated that motivation can attenuate the cognitive declines that arise as a result of sleep deprivation over short durations of time^14^. However, most studies have investigated responses to extrinsic motivators over acute sleep deprivation (i.e., when financial incentives were given to participants), which may not be consistent with the influence of intrinsic motivation (i.e., astronauts driven to succeed in a mission)^15,16^. Intrinsic motivation is modulated according to time awake and circadian phase, with a peak in motivation occurring during the biological day^17^, suggesting that individuals who are working during the day and only modestly sleep deprived may be able to overcome some performance impairment when they are intrinsically motivated to perform well. Given the mixed findings on measures of performance during spaceflight, it remains unclear whether individuals selected to be astronauts can maintain performance over the course of a mission via intrinsic motivation alone to succeed even when sleep restricted.

Several spaceflight simulations have been conducted previously; however, the study schedules for most prior spaceflight simulations were not designed to evaluate the performance of crewmembers during sleep restriction. For example, two Mars mission simulations lasting 105 and 520 days showed that crewmembers experienced reduced performance throughout the missions, but crewmembers received 8-h sleep opportunities on most days and both crews averaged over 7 h of sleep per night during those missions^2,18^. These findings suggest that even modest sleep loss affects performance among rigorously selected individuals, yet it is unclear how shorter mission durations, such as those that are consistent with what astronauts will experience during lunar exploration, might influence performance.

Earth-based studies of young, healthy individuals suggest that there is a dose-response relationship between sleep loss and performance, in that there is an immediate progressive reduction in performance with every day of sleep less than 8 h^19–21^. In addition, such studies find that people are unable to estimate the magnitude of their performance impairment on average after several days of sleep restriction^19^. This means that individuals experiencing chronic sleep restriction do not recognize that they are performing poorly. In operational environments where there are few individuals available to complete tasks and where individuals are highly motivated, this disconnect could increase the potential for an operational error.

The mismatch between how individuals feel and how they perform has been recognized in many safety-sensitive industries. For example, in aviation operations, pilots are required to self-report that they are fit-for-duty, but this self-report is supplemented by schedule optimization using bio-mathematical models^22^. These models are used by scheduling personnel to identify schedules that would place individuals at work during a vulnerable period, when sleep loss and circadian misalignment may cause performance degradation. It is conceivable that such models could be used by astronauts to self-schedule tasks, thereby avoiding the need to rely on operational personnel to monitor crew and reassign tasks. Given the uncertainties associated with how highly-motivated individuals perform during sleep restriction, it is unclear whether currently available models would be appropriate for this purpose.

Considering the lack of information on how crewmembers may be expected to perform during moderate-duration lunar missions, we aimed to determine how rigorously selected, astronaut-like individuals participating in a simulated spaceflight mission would perform during chronic sleep restriction. We further aimed to evaluate how well bio-mathematical models designed to predict performance changes due to sleepiness would correlate with actual performance in an operational environment.

## Methods

### Participants

Participants were solicited through advertisements at NASA, various military groups, academic institutions, and among the general public through https://www.nasa.gov/analogs/hera and were selected to be “astronaut like.” Participants were required to be non-smokers, 30–55 years of age, have English language proficiency, and at least a Master of Science in a science, technology, engineering, mathematics (STEM) discipline or the equivalent years of experience. Volunteers were required to meet the NASA long-duration space flight physical standards, which were verified by physical exam. Exclusion criteria were a body mass index (BMI) greater than 30, height greater than 74 inches, any history of sleep disorders or regular use of sleep aids, any chronic health conditions, regular medication use or had dietary restrictions that could not be stopped during the mission. Individuals were selected to populate five separate crews of four crewmembers each.

### Human Analog Research Exploration (HERA) habitat

**Figure 1:**
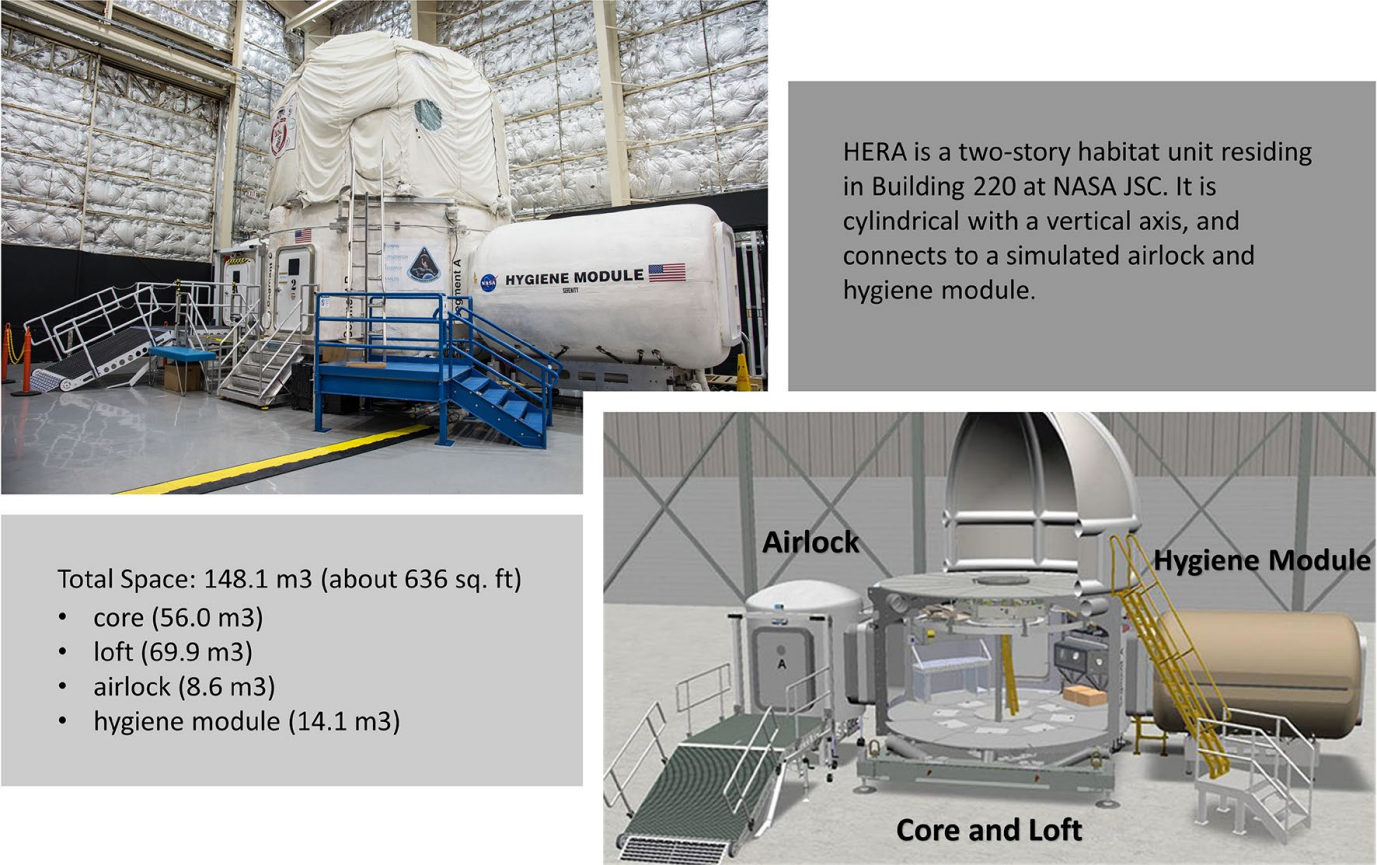
HERA habitat (https://www.nasa.gov/analogs/hera).

The HERA habitat is a two-story unit that has an airlock, a hygiene module, and crew quarters (Fig. 1). The habitat is approximately 636 sq. ft., distributed throughout the modules. The habitat contains spaceflight simulation workstations, a galley, a communication station, an aerobic exercise station, and private sleep quarters. The habitat room temperature was maintained at 72 °F (± 5 °F), with 70% (± 10%) humidity for all missions. All necessary equipment, materials, food, and supplies for four crew members to survive inside the habitat for 45 days was provided.

### Pre-mission procedures

Participants were oriented to the habitat and completed a week of training on mission procedures prior to ingress. During this time, they also provided demographic information and completed questionnaires describing their baseline characteristics.

### Mission schedule and procedures

**Figure 2:**
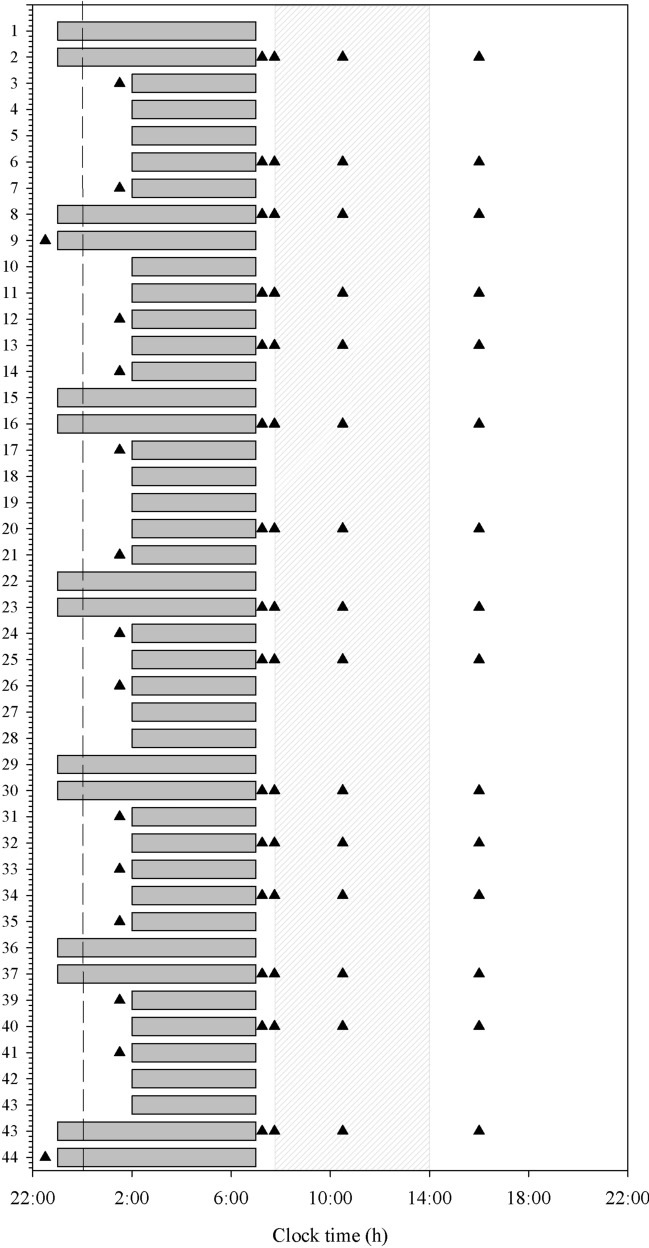
Study schematic for the 45-day protocol. Gray bars indicate schedule sleep. Triangles indicate the days and timing of psychomotor vigilance tests PVT. Hatched region indicates the duration of time when crewmembers were allowed to consume caffeine. The vertical dashed line indicates midnight.

Five missions were scheduled for HERA Campaign 4. Each mission was scheduled for 45 days and followed the same study schedule. Crewmembers were monitored continuously by a simulated mission control to ensure their health and well-being. All activities were prescheduled and included simulated operational tasks, such as extra vehicular activities, robotic arm manoeuvres, and science activities. The crew were not required to follow any specific sleep schedule prior to entering the habitat. During the 2 weeks prior to the mission, participants completed a sleep diary that was used to estimate pre-study sleep duration. During the mission, crewmembers were scheduled for 5 h of sleep during the week (5 nights) and 8 h of sleep on the weekends (2 nights; Fig. 2). Napping was not allowed during the protocol and crewmembers were monitored by mission control staff to verify their adherence to study procedures. Caffeine was only allowed between 07:45 and 14:00 h. Timing and amount of caffeine consumption was not documented.

Crewmembers completed a 5-min version of the psychomotor vigilance task (PVT) five times a day on 15 days distributed throughout the mission (approximately every three days). The PVT sessions were scheduled: (1) within 30 min of waking, (2) 30 min after the first PVT, (3) mid-morning, (4) mid-afternoon, (5) before going to bed. Following the first PVT of the day, the crewmembers were instructed to complete the Samn Perelli rating of fatigue.

### Bio-mathematical models

We compared the predictions of four bio-mathematical models to the actual PVT data collected in the missions. These models included (1) the Adenosine-Circadian model, which combines the Harvard Circadian Performance model with a physiological model based on the action of adenosine, which simulates the effects of chronic sleep restriction on performance^23–29^; (2) the Unified Model of Performance, which was developed to combine the effects of total and chronic sleep restriction^30^; (3) the Washington State University (WSU) State-space model, which uses coupled, non-homogeneous first-order ordinary differential equations to account for changes in performance associated with chronic sleep restriction^31,32^; and (4) the Sleep, Activity, Fatigue, and Task Effectiveness (SAFTE) model, which is based on the two-process model of sleep regulation and was originally developed for the US Army and Air Force^33,34^. These models were chosen because they were either funded in-part by NASA or used for other US government or military operations similar to spaceflight missions.

#### Model input and output

We used the interfaces developed by the modelling groups (when available) in order to assess the usability of the interfaces that would be used by operational personnel. We used the commercially available Fatigue Avoidance Scheduling Tool (FAST) to generate predictions for the SAFTE model^35^ and the web-based 2B-Alert interface to generate predictions for the Unified model^36^. Dr. Hans Van Dongen provided us with a DOS-executable interface for evaluation of the WSU State-space model, and Dr. Andrew Phillips provided the equations used to generate predictions for the Adenosine-Circadian model in MATLAB.

The methods for inputting each model differed based on the interface available for the input of data and by the assumptions made by the model. Table 1 shows the possible model inputs, the inputs that we used, and the model outputs.

**Table 1:** Description of model interface, input values, input data, and output.

Model	Interface	Input Values	Input Data	Output(s)
Adenosine-circadian	Equations programmed in MATLAB	Time, light levels, scheduled sleep	Sleep schedule, mission light levels	PVT Lapses
State-space	DOS-executable interface	Time, scheduled sleep	Sleep schedule	PVT Lapses
Unified	Web-based interface	Time, scheduled sleep, caffeine dose and time	Sleep schedule	PVT Lapses, PVT reaction time, PVT response speed
SAFTE FAST	Software program	Time, scheduled sleep, work schedule	Sleep schedule	Cognitive effectiveness^a^

^a^Cognitive effectiveness is a PVT-based measure.

### Data analysis

#### Psychomotor vigilance task

Five PVT outcomes were considered in overall analyses: (1) mean reaction time (RT), (2) response speed (calculated as (1/mean RT) × 1,000), (3) lapses (count of RT > 500 ms), 4) mean RT of 10% fastest responses (10% fastest RT), (5) mean RT of 10% slowest responses (10% slowest RT). These outcomes were selected because they have previously been shown to be associated with sleep loss and impaired performance^37^. We used PVT lapses as our primary measure to compare to the bio-mathematical model predictions because three of the four models provide estimated lapse values as their predicted performance output.

#### Samn Perelli

The Samn Perelli ratings of fatigue were completed once per day, in conjunction with the first PVT of the day.

#### Scaling procedures

A challenge in comparing model predictions to actual performance measures is that the model outputs differ from the objective metrics available for comparison. For example, the SAFTE model, which outputs the proprietary measure “cognitive effectiveness,” could be compared to objective data, but the results of such a comparison would not be on the same scale as the other models, making it impossible to compare the model predictions to one another. In addition, although we used PVT data to assess waking performance, we used a 5-min version of the PVT. The PVT lapse predictions for each model are based on a 10-min test. As a result, comparisons between the model predictions and actual data produced by crewmembers was expected to yield consistent overestimation of PVT lapses relative to actual lapses. Due to all of these factors, we used a least-squares-optimal scaling approach to scale the model outputs relative to the data as described by Van Dongen, using PROC NLMIXED in SAS (SAS Institute Inc. Cary, NC)^38^.

### Statistical comparisons

#### Performance and fatigue

We evaluated changes in the Samn Perelli and PVT performance over the course of the mission and by 8-h versus 5-h sleep condition using linear mixed-effects regression models, including participant as a random factor (PROC MIXED in SAS), and adjusted for pre-mission sleep duration. We used the same approach to evaluate PVT outcomes by test session of the day. For these models, we allowed the slopes and intercepts to vary by individual participant.

#### Comparison between actual and predicted performance

We used all of the data available for each individual to compare to the scaled predictions for each model to the observed data. In order to assess agreement for each model to the actual data, we generated repeated measures correlation estimates in the statistical software program R (R: A Language and Environment for Statistical Computing, R Core Team, R Foundation for Statistical Computing, Vienna Austria, 2019, https://www.R-project.org) using the rmcorr package. In order to compare the model predictions to each other, we calculated the root mean square error (RMSE) and the normalized root mean square error (NRMSE) by dividing the RMSE value by the mean of the observed.

In order to evaluate the ability of each model to predict group-average performance over time, we used linear mixed-effects models, using random effects intercepts for participant and mission, to obtain R-square values as a metric of agreement and evaluate the slope and intercept coefficients of the model.

Further, to visualize the observed data and scaled model predictions by day of mission, session of day, and by sleep condition (8 h vs. 5 h of sleep), we used mixed models to obtain point estimates and confidence intervals of the means for each test by time, day, and sleep condition. These means and confidence limits can be compared to determine under which conditions (time or sleep schedule) the models predict closer or further from the observed.

### Ethics declarations

All participants provided informed consent prior to engaging in any study procedures. All study procedures conformed to the guidelines set forth in the United States Common Rule. This study was approved by the NASA Johnson Space Center Institutional Review Board (protocol PRO2328).

## Results

Sixteen participants completed four HERA missions (n = 6 female) and an additional four individuals completed 20 days of one mission (n = 1 female). The second mission of Campaign 4 was aborted due to a hurricane in the vicinity of the habitat that threatened the safety of the crew and support staff. Data from all five missions were used in analyses, except where indicated. Demographic characteristics for all participants are shown in Table 2.

**Table 2:** Demographic information for all HERA crewmembers.

Variable	M (SD)	Range
Age	38.65 (8.19)	30–55
BMI	24.39 (2.90)	19.37–29.30
Pre-study sleep duration (h)	6.85 (0.64)	5.6–7.8
Pre-study bedtime	23:43 (1:18)	22:28–01:38
Pre-study waketime	06:33 (0:52)	05:29–07:15
Beck Depression Inventory	1.62 (2.06)	0–7
MEQ	52.85 (12.32)	26–73
**STAI**		
State Anxiety Score	48 (3.80)	39–53
Trait Anxiety Score	45.6 (3.32)	38–50

Sleep duration was self-reported in a sleep diary during the 2 weeks before the mission.

*BMI *body mass index, *h *hours, *MEQ *Morningness-Eveningness questionnaire, *STAI *State-Trait Anxiety Inventory.

### In-mission performance

#### Fatigue and performance by day of mission

**Figure 3:**
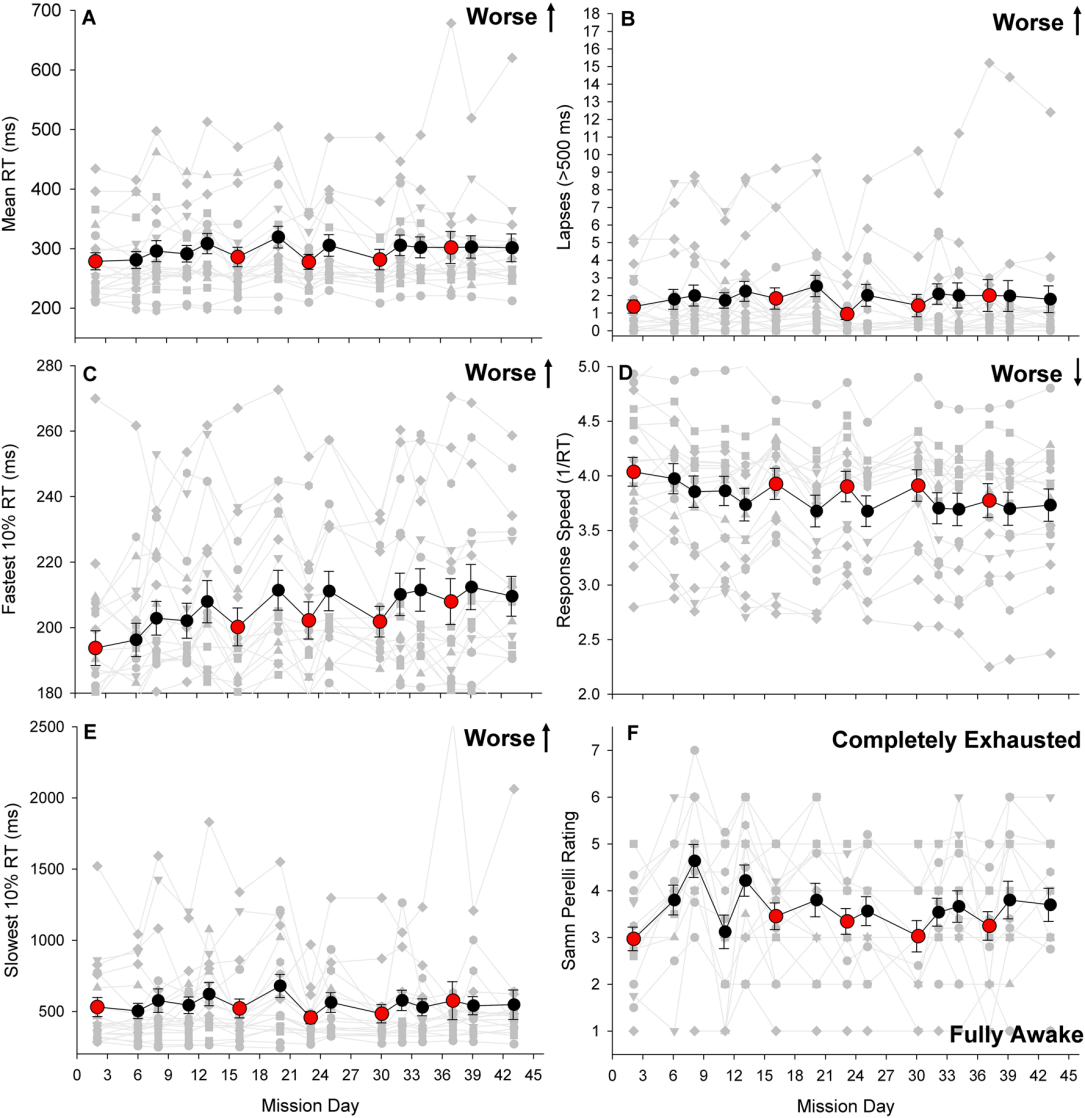
Average (black and red circles) psychomotor vigilance task (PVT) performance and individual daily mean values (light gray circles) by mission day for mean reaction time (**A**), lapses > 500 ms (**B**), fastest 10% reaction time (**C**), response speed (**D**), slowest 10% reaction time (**E**), and Samn Perelli ratings (**F**) by day of mission. Note differences in y-axis scale for mean, fastest and slowest reaction times. Black circles indicate days following 5 h of sleep, red circles indicate days following 8 h of sleep. *RT *reaction time, *ms *milliseconds, error bars reflect the standard error of the mean.

We evaluated fatigue ratings and PVT reaction time (Fig. 3A), lapses (Fig. 3B), fastest 10% reaction time (Fig. 3C), response speed (Fig. 3D) and slowest 10% reaction time (Fig. 3E) over the course of the mission for all participants combined. We found a significant worsening of performance from the beginning to the end of the mission for mean reaction time, response speed, and fastest 10% reaction time (all p < 0.01). We did not find a significant increase in lapses over the course of the mission (p = 0.14), nor did we observe a significant reduction in performance as measured by the slowest 10% reaction time (p = 0.91). However, the slowest 10% reaction time was elevated (mean > 500 ms) from the beginning of the mission, suggesting that the participants were already sleep deprived early in the mission. Self-reported fatigue ratings did not show a linear decline over the course of the mission (p = 0.57; Fig. 3F).

There were large differences in the performance trajectories of the individual crewmembers, with some individuals maintaining stable performance throughout the mission and others exhibiting a large decline in performance over the course of the mission (Fig. 2, gray shaded lines). To further evaluate these differences, we stratified the group by tertile of average in-mission PVT performance. The performance between the best and worst tertile groups was similar in trajectory across the mission (supplemental material and Figure S1).

#### Fatigue and performance by sleep condition

**Figure 4:**
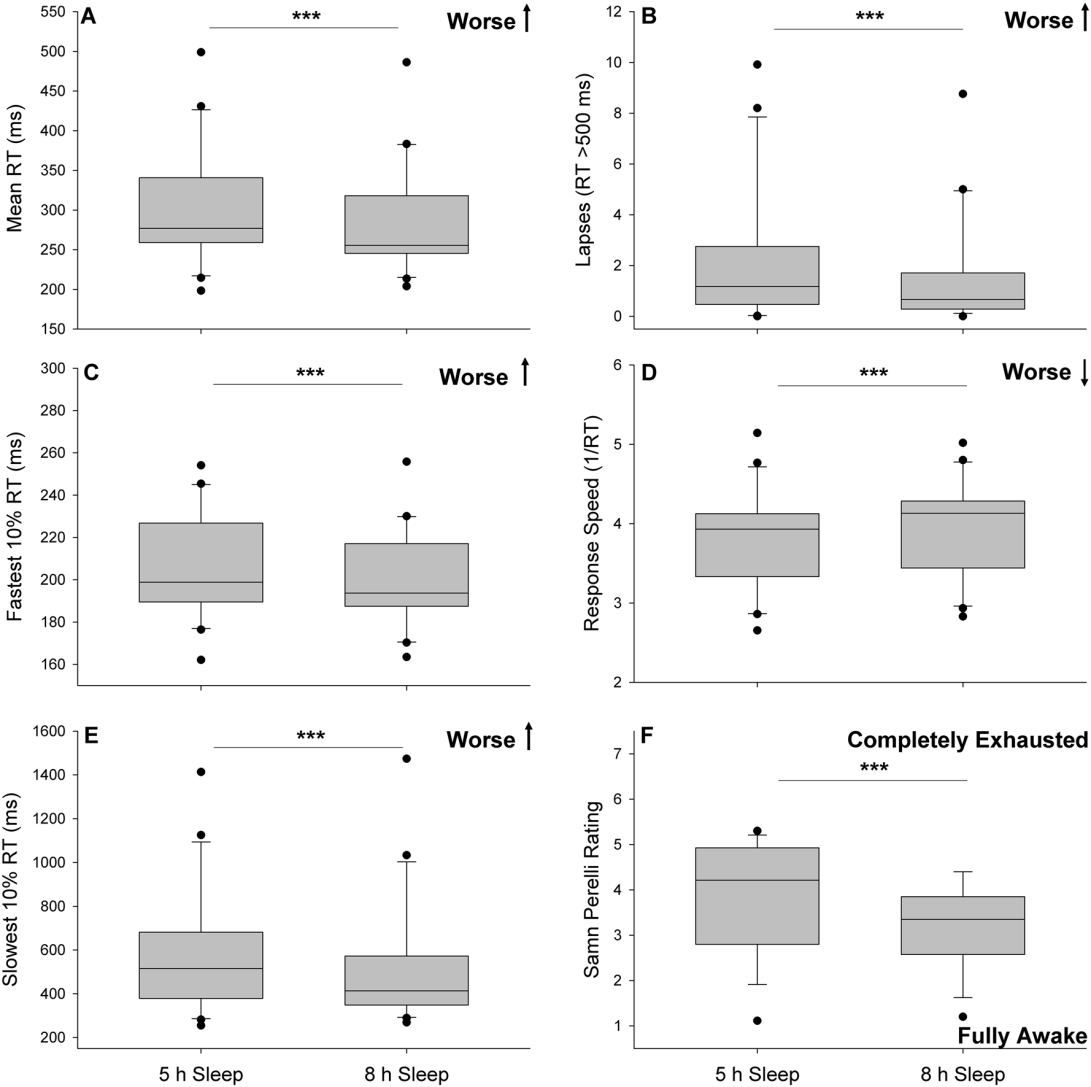
Average psychomotor vigilance task (PVT) performance for mean reaction time (**A**), lapses (**B**), fastest 10% reaction time (**C**), response speed (**D**), slowest 10% reaction time (**E**), and Samn Perelli ratings (**F**) by prior night’s sleep duration. Note differences in y-axis scale for mean, fastest and slowest reaction times. *RT *reaction time, *ms *milliseconds. ***p < 0.01.

We found that all PVT outcomes were poorer following 5 h of sleep, relative to following 8 h of sleep (all p < 0.01; Fig. 4A–E) We further found that self-reported fatigue was significantly greater on days following 5 h of sleep compared to days following 8 h of sleep (p < 0.001; Fig. 4F). These findings were similar for those in the worst tertile of performance; however, there was no significant difference in performance among those in the tertile of best performers (supplemental material; Figure S2).

#### Performance by session-of-day

**Figure 5:**
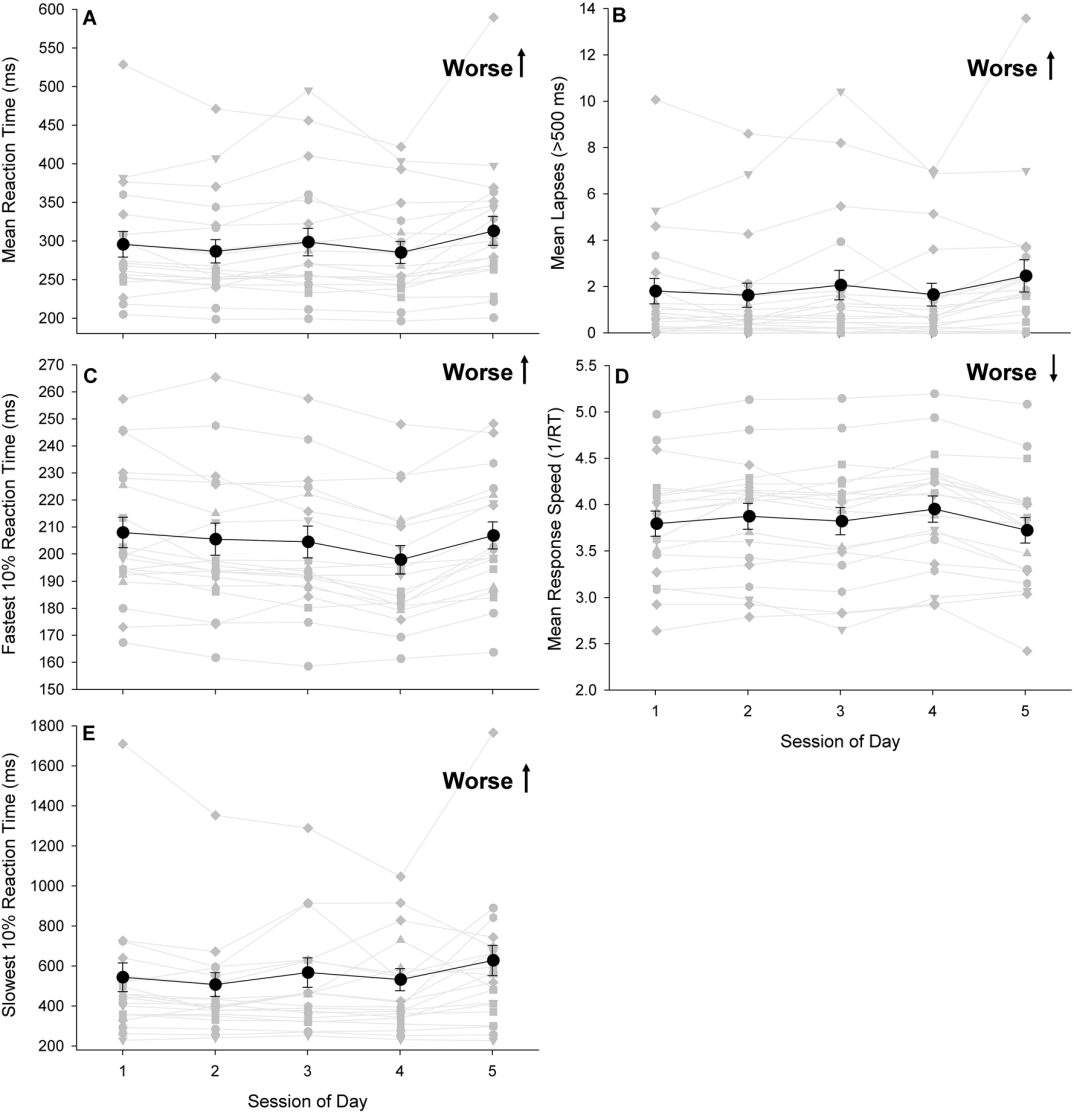
Average (black circles) psychomotor vigilance task (PVT) performance and individual (light gray circles) mean performance by session for mean reaction time (**A**), lapses (**B**), fastest 10% reaction time (**C**), response speed (**D**), and slowest 10% reaction time (**E**) by session of the day. Note differences in y-axis scale for mean, fastest and slowest reaction times. *RT *reaction time, *ms *milliseconds, error bars reflect the standard error of the mean.

We evaluated PVT reaction time, response speed, lapses, fastest and slowest 10% reaction time by the session of the day, averaged over sessions from all days of the mission. We found that lapses significantly worsened (p = 0.048) from the beginning to the end of the day (Fig. 5B). Mean PVT RT (p < 0.12), slowest 10% RT (p = 0.07), and response speed did not change significantly by session of the day (p = 0.99; Fig. 5 A, D, and E) and fastest 10% RT significantly improved from the beginning to the end of the day (p = 0.02; Fig. 5C).

#### Association between model predictions and actual performance by day of mission

**Figure 6:**
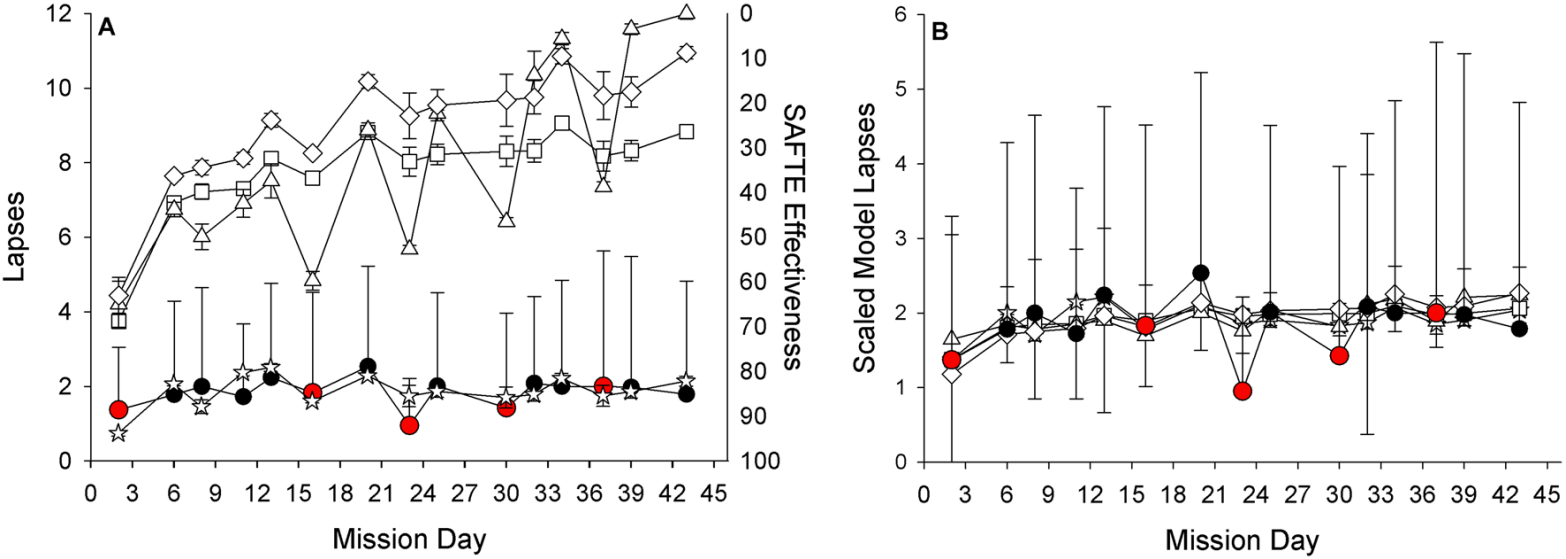
Relationship between model predictions (**A**) and scaled model predictions (**B**) and associated confidence intervals with actual lapses (± standard error) by day of the mission. Actual performance measures are shown as filled circles, model predictions are shown as open symbols as follows: triangles = adenosine-circadian model, squares = unified model, diamonds = state-space model, stars = SAFTE model. Black circles indicate days following 5 h of sleep, red circles indicate days following 8 h of sleep. Note, SAFTE model outcome “cognitive effectiveness” ranges from 0–100 and is plotted in the inverse on panel (**A**).

As expected, the raw model lapse predictions were higher than the observed group-average lapse values throughout the mission (Fig. 6A). The linear mixed-effects regression point estimates and confidence intervals for the scaled model predictions relative to the observed lapse values for each day of the mission suggests that the scaled models were able to predict lapses by day of the mission on average (i.e., the observed data is within the confidence intervals for the model predictions for most models on most days; Fig. 6B).

#### Association between model predictions and actual performance by session of day

**Figure 7:**
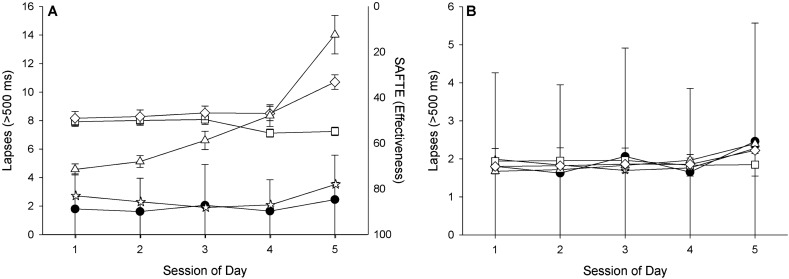
Relationship between model predictions (**A**) and scaled model predictions (**B**) and associated confidence intervals with actual lapses (± standard error) by session of the day for all days combined. Actual performance measures are shown as filled circles, model predictions are shown as open symbols as follows: triangles = adenosine-circadian model, squares = unified model, diamonds = state-space model, stars = SAFTE model. Note, SAFTE model outcome “cognitive effectiveness” ranges from 0–100 and is plotted in the inverse on panel (**A**).

**Figure 8:**
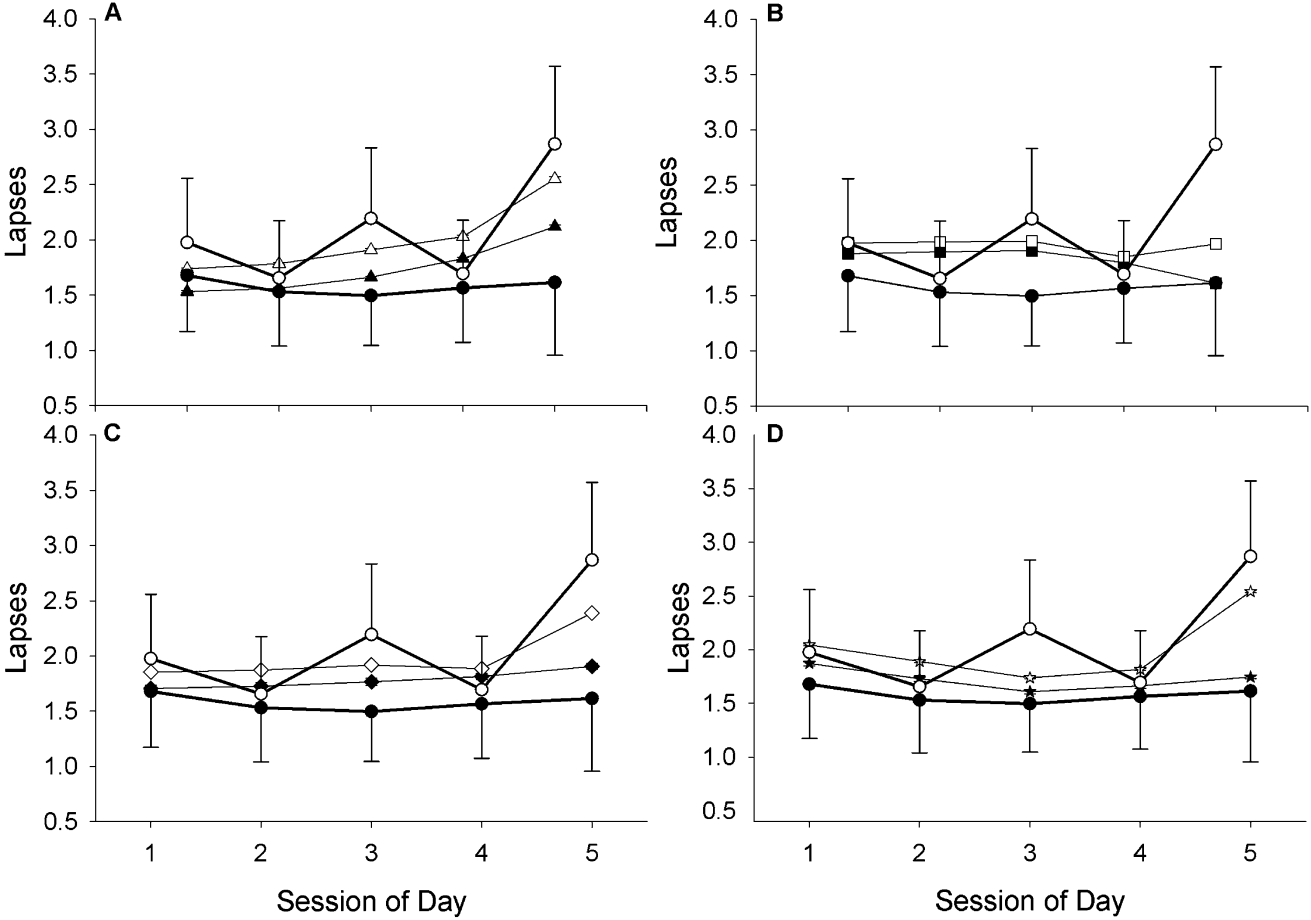
Relationship between scaled model predictions and actual lapses (± standard error) by session of the day following 8 h sleep (filled symbols) and 5 h of sleep (open circles). Actual performance measures are shown as circles (filled for 8-h, open for 5-h), model predictions are shown as follows: triangles = adenosine-circadian model (**A**), squares = unified model (**B**), diamonds = state-space model (**C**), stars = SAFTE model (**D**). Note, confidence intervals are narrow for the model predictions and are not visualized on the plot.

The raw model lapse predictions were higher than the observed group-average lapse values by session of the day (Fig. 7A). The linear mixed-effects regression point estimates and confidence intervals for the scaled model predictions relative to the observed lapse values suggests that the scaled models were able to predict lapses by session of the day on average (Fig. 7B). When we stratified the session of the day data by days following 8 h of sleep compared to days following 5 h of sleep, we found that the scaled models were generally able to capture the average changes in performance over the course of the day (Fig. 8A–D), although on days following 5 h of sleep, the midday data collection (session 3) and the pre-bedtime data collection (session 5) fell outside of the confidence intervals for all of the model predictions.

#### Overall goodness of fit measures between model predictions and actual performance

Each of the models yielded a weak correlation with the actual data overall according to the repeated measures correlation for scaled lapses (Table 3).

**Table 3:** Root mean square error (RMSE) values (lower is better), normalized RMSE (NRMSE) values (lower is better), and repeated measures correlation results for the scaled model predictions relative to the actual lapse values for each crewmember.

	RMSE	NRMSE	Repeated measures correlation	p-value
Adenosine-circadian (scaled lapses)	9.19	4.98	0.17	< 0.01
Unified (scaled lapses)	9.31	5.04	0.10	< 0.01
State-space (scaled lapses)	9.25	5.01	0.17	< 0.01
SAFTE (scaled lapses)	9.22	4.99	0.18	< 0.01

The normalized-RMSE (NRMSE) scores allow for comparison between the models, with a lower NRMSE value indicating better performance by the model. The NRMSE values were similar for all of the models (Table 3).

We compared models to the data for days following 8-h sleep episodes and for days following 5-h sleep episodes. The models performed worse on average for days following the 8-h sleep episodes (Table 4) than for the days following the 5-h sleep episodes (Table 5) according to the repeated measures correlation. As with the overall comparison, the NRMSE scores were similar for all models following nights with 8 h of sleep compared to nights with 5 h of sleep, suggesting that no specific model was superior in this stratified analysis.

**Table 4:** Root mean square error (RMSE) values (lower is better), normalized RMSE values (lower is better), and repeated measures correlation results for the scaled model predictions relative to the actual lapse values for each participant for the 8-h sleep condition.

	RMSE	NRMSE	Repeated measures correlation	p-value
Adenosine-circadian (scaled lapses)	7.86	5.11	0.02	0.69
Unified (scaled lapses)	7.83	5.09	0.04	0.43
State-space (scaled lapses)	7.82	5.08	0.05	0.36
SAFTE (scaled lapses)	7.68	4.99	0.07	0.69

**Table 5:** Root mean square error (RMSE) values (lower is better), normalized RMSE values (lower is better), and repeated measures correlation results for the scaled model predictions relative to the actual lapse values for each participant for the 5-h condition.

	RMSE	NRMSE	Repeated measures correlation	p-value
Adenosine-circadian (scaled lapses)	9.84	4.93	0.17	< 0.01
Unified (scaled lapses)	10.03	5.02	0.09	< 0.01
State-space (scaled lapses)	9.95	4.98	0.19	< 0.01
SAFTE (scaled lapses)	9.97	4.99	0.18	< 0.01

## Discussion

We found that rigorously selected, astronaut-like individuals experienced a progressive reduction in performance over the course of a simulated space mission that included chronic sleep restriction. Crewmembers also performed worse on days following 5 h of sleep relative to days following 8 h of sleep. In our comparison of four bio-mathematical models relative to actual in-mission performance, we found that all the models were able to capture relative changes in performance by day of mission and by session of the day but performed less well at capturing the differences in performance over days following 5 h of sleep. Although the models performed similarly to one another, they were only weakly correlated to the observed data according to our goodness of fit assessments. Our findings have implications for future space missions and for other occupations comprised of rigorously selected populations that must maintain a high level of performance despite persistent chronic sleep restriction.

Our findings suggest that simply meeting the criteria required to be an astronaut is not in itself a determinant of resilience to chronic sleep restriction. This finding is consistent with data from populations of high-performing professionals such as physicians, airline pilots, and special operations military units, who are often required to work extended-duty work shifts that limit sleep opportunity, resulting in performance impairment^39–41^. The population of rigorously selected individuals that we studied exhibited performance impairment over the course of a 45-day simulated space mission on average. Notably, there were large individual differences in tolerance to the sleep restriction schedule, with some crewmembers performing well throughout the mission and with others exhibiting concerning levels of performance impairment within the first week of the mission. Some of the crewmembers reported short sleep duration pre-mission, which may explain why some individuals exhibited poor performance early in the study. Given that the crew were only afforded 8 h of sleep opportunity during their “long” sleep nights, it is likely that those who were sleep deprived at baseline never had the opportunity to recover during the mission where they accumulated further sleep debt.

Interindividual differences in tolerance or vulnerability to sleep loss have been found in other laboratory and field studies, including fighter pilots and public safety workers^42–44^. Further research is needed to determine whether these differences in performance relate to differences in intrinsic motivation, differences in sleep need, or resilience to sleep loss. Despite the overall change in performance that we observed over the course of the mission, we did not observe differences in Samn Perelli fatigue ratings. Crewmembers rarely rated themselves as completely exhausted (SP rating of 7), but they did rate themselves as relatively more fatigued following nights with 5 h of sleep relative to nights with 8 h of sleep. Such discrepancies between subjective and objective measures of sleepiness have been documented in other settings, including long-haul international aircrew^45^. Our findings suggest that it is important to evaluate individuals in safety sensitive occupations using objective measures of performance in order to determine how an individual is affected by sleep loss.

NASA plans to send astronauts to orbit the Moon by 2022, with the first lunar exploration mission to occur in 2024. During these missions, crewmembers will be exposed to a high-tempo workflow involving challenging mental and physical workload while establishing the lunar Gateway and habitats. As a result, it is critical that crewmembers maintain alertness and cognitive performance during all phases of these missions. Crewmembers average approximately 6 h of sleep during spaceflight^46^, but there have been few attempts to characterize changes in performance that may accompany the short sleep duration that astronauts experience in space. In two case studies of cosmonauts, and in one study of four astronauts, no significant decrements in performance were observed during short-duration missions relative to ground-based measures^8,11,13^. In contrast, two separate studies aboard the Space Shuttle (of three and five astronauts) found that crewmembers experienced impaired performance during spaceflight, changes which the authors attributed to fatigue and sleep loss among the crew^3,10^. More recent evidence of performance impairment during spaceflight suggest that such changes may relate to a global increase in local sleep-like events during spaceflight compared to on Earth^9^, providing evidence for a relationship between sleep deficiency and performance impairment during spaceflight. Although our data were collected in a spaceflight analogue environment, they suggest that sleep loss during future space missions can be expected to impair performance even among a rigorously selected crew.

NASA currently provides crewmembers with an 8-h sleep opportunity during spaceflight missions, yet crew still average 6 h of sleep per night. It may be necessary to provide crew with additional tools to promote longer sleep, such as providing crew with stable schedules to minimize circadian misalignment^46^ and sleep quarters that minimize environmental disruption^47^. If such measures are insufficient for extending crew sleep, then countermeasures such as scheduled napping, strategic use of caffeine, and other alertness and sleep-promoting medications may be necessary to ensure the crew are fit for duty. Bio-mathematical models may provide some utility in helping crewmembers determine when to use such countermeasures.

In our comparisons, we found that all of the models performed similarly to one another, suggesting that no single model is superior to the others. We found that all models appeared to be able to predict group-average performance by day of the mission and by session of the day on average. When we examined session of the day by prior night’s sleep duration (8-h vs. 5-h), we found that the observed performance was worse midday and before bed relative to the model predictions following 5 h of sleep. The worse performance that we observed during the midday session could relate to a metabolic response following lunch (the so-called ‘post-lunch dip’)^48^. It is also possible that this poorer performance relates to the cessation of caffeine, which crewmembers were not allowed to use after 14:00 h. None of the models include information on how metabolism may influence performance. Only the Unified Model of Performance allows for the input of caffeine^49,50^, but the timing and amount of caffeine use during the mission was not recorded in a way that we could include in the model. It is likely that the predictions of the Unified model would be improved with the addition of such information. The poorer performance exhibited by the participants at the end of the day may relate to the build-up of chronic sleep restriction over time, potentially interacting with the circadian rhythm promoting sleepiness. This is consistent with the findings of St. Hilaire et al. (2016), who evaluated the same models and showed that none of the models were able to capture the accumulation of chronic sleep debt that accrued over three cycles of two nights with 3 h of sleep followed by a 10-h recovery night^29^. Although, in that study, the models consistently underestimated performance at all points during the day on the second day of the third cycle of sleep restriction (i.e., the average performance in St. Hilaire et al. was much worse than what we observed). It is possible that these differences stem from the differences in the sleep opportunities afforded to participants in our studies (3 h for 2 nights/10 h recovery for 1 night vs. 5 h for 5 nights/8 h recovery for 2 nights).

Our goodness of fit analyses found that the models were only weakly correlated with the actual data. The poor correlations that we observed are likely the result of individual differences in response to chronic sleep restriction. As a result, it would be advisable to use models such as those that we evaluated for general scheduling purposes (i.e., to determine if one schedule is superior to another) and not for making decisions about individual fitness for duty. In the spaceflight environment it could be risky for a vulnerable individual to be provided with average-level performance predictions, which might lead to that crewmember engaging in a dangerous task while unknowingly in a compromised state. There are models other than those that we evaluated that have been designed to determine individual changes in performance in response to sleep loss^51^. Such individualized models have the potential to allow for a personalized medicine approach to determining an individual’s fitness for duty and should be evaluated to determine their feasibility for operational implementation.

Although we were able to collect systematic information from carefully selected, astronaut-like individuals, our study is not without limitation. Our assessment was conducted in an Earth-based habitat that simulated the conditions of spaceflight; however, microgravity itself may influence cognition and the amount of sleep a crewmember requires in space. Despite this, our findings support the notion that individuals who possess the ‘right stuff’ are not immune to the effects of sleep loss on Earth. In addition, we used the PVT as our primary measure of cognitive impairment. The PVT is highly sensitive to sleep loss, but poor performance on the PVT may not reflect how crewmembers would perform on the more complex tasks required during a space mission^52^. Finally, while crewmembers were restricted to 5 h of sleep during the week and 8 h of sleep on the weekends, we did not have objective sleep data. Although it is unlikely that the crewmembers achieved more sleep than scheduled, because they were prohibited from napping and were continuously monitored by mission control personnel. It is possible that the crew obtained less sleep than their time in bed, but we expect that the difference between time in bed and sleep duration would be modest given the sleep restriction protocol.

In this study we found that rigorously selected, astronaut-like individuals are susceptible to the effects of chronic sleep restriction. On average, crewmembers in this simulated spaceflight mission experienced a progressive decline in performance over 45 days of sleep restriction and they performed worse at the end of the day relative to the beginning of the day, although some individuals appeared able to sustain performance over the course of the mission, while others appeared more vulnerable. We found that bio-mathematical models designed to predict changes in performance following sleep loss may be useful tools for designing schedules for future space missions, but individualized models should be evaluated for potential fitness for duty application. Our findings support the concern that chronic sleep loss is associated with performance impairment during spaceflight and suggest that measures should be taken to ensure that crewmembers are provided with the schedules and environment necessary for optimal duration and quality of sleep.

## Supplementary information

Supplementary Information.

## Data Availability

The datasets generated and analysed during the current study are available by request from the NASA Life Sciences Data Archive: https://lsda.jsc.nasa.gov
