# Supplementary material for: Changes in performance and bio-mathematical model performance predictions during 45 days of sleep restriction in a simulated space mission
Source: Sci Rep. 2020 Sep 24;10:15594. doi: 10.1038/s41598-020-71929-4 (PMC7515915; doi:10.1038/s41598-020-71929-4)
Supplement: Supplementary file 1 — Supplementary Information. [file 41598_2020_71929_MOESM1_ESM.docx]

**Changes in Performance and Bio-mathematical Model Performance Predictions during 45 Days of Sleep Restriction in a Simulated Space Mission**

Erin E. Flynn-Evans PhD, MPH,^1*^ Crystal Kirkley MS,^2^ Millennia Young PhD,^3^ Nicholas Bathurst MA,^2^ Kevin Gregory,^1^ Verena Vogelpohl,^4^ Albert End MS,^4^ Steven Hillenius MHCI,^5^ Yvonne Pecena PhD,^4^ Jessica J. Marquez PhD^5^

^1^ NASA Ames Research Center, Fatigue Countermeasures Laboratory, Human Systems Integration Division, Moffett Field CA, 94035

^2^ San José State University Research Foundation, Fatigue Countermeasures Laboratory, Human Systems Integration Division, Moffett Field CA, 94035

^3^ NASA Johnson Space Center, Biomedical Research and Environmental Sciences Division

Human Health and Performance Directorate, Houston TX,

^4^ German Aerospace Center (DLR), Department of Aviation and Space Psychology, Hamburg Germany

^5^ NASA Ames Research Center, Human Computer Interaction Group, Human Systems Integration Division, Moffett Field CA, 94035

*Corresponding author:

Erin Flynn-Evans

NASA Ames Research Center

Fatigue Countermeasures Laboratory N262-4

Moffett Field CA 94035

e-mail: erin.e.flynn-evans@nasa.gov

**Supplemental Material**

*Fatigue and performance by day of mission among the best and worst tertile*

In order to further explore differences in performance, we stratified the participants into tertiles to evaluate the best (n = 6) and worst (n = 7) performers based on their average PVT performance during the mission. We used linear mixed effects models with participant as a random effect to evaluate changes in performance over the course of the mission. Changes in performance over the mission were similar for each group, with both the best and worst groups exhibiting slowing response speed and fastest 10% reaction time over the course of the mission (p < .01 for all; Figure S1). Mean reaction time (p = .10, p = .10), lapses (p = .64, p = .06), slowest 10% reaction time (p = .25 and p = .49), and Samn Perelli ratings (p = .97, p = .28) did not show statistically significant changes over time in mission among the best and worst groups respectively. However, due to the small sample size in each group, it is possible that we were underpowered to detect changes in performance.

*Fatigue and performance by sleep condition among the best and worst tertiles*

We found that all PVT outcomes were poorer following five hours of sleep, relative to following eight hours of sleep for the worst tertile (mean reaction time p < .01, lapses p = .02, fastest 10% reaction time p = .01, response speed p < .01, slowest 10% reaction time p = .05; Figure S2). In contrast, the best tertile did not exhibit differences in performance following five hours of sleep relative to eight hours of sleep (mean reaction time p = .19, lapses p = .32, fastest 10% reaction time p = .12, response speed p = .22, slowest 10% reaction time p = .29; Figure S2). Neither group showed significant differences in self-reported fatigue on days following eight hours of sleep relative to five hours of sleep. Although we may be underpowered to detect differences in performance in the sleep conditions among the best tertile, our findings suggest that some of the crewmembers may have been resilient to the effects of sleep loss. These findings should be explored further in larger cohorts in order to determine whether such differences are traits that could be identified during the crew selection process.

**Figure S1.** Average psychomotor vigilance task (PVT) performance for the worst (red circles) and best (green circles) tertile of participants by mission day for mean reaction time (A), lapses > 500 ms (B), fastest 10% reaction time (C), response speed (D), slowest 10% reaction time (E), and Samn Perelli ratings (F) by day of mission. Note differences in y-axis scale for mean, fastest and slowest reaction times. RT = reaction time, ms = milliseconds, error bars reflect the standard error of the mean.

**Figure S2.** Average psychomotor vigilance task (PVT) performance for mean reaction time (A), lapses (B), fastest 10% reaction time (C), response speed (D), slowest 10% reaction time (E), and Samn Perelli ratings (F) by prior night’s sleep duration. Note differences in y-axis scale for mean, fastest and slowest reaction times. RT = reaction time, ms = milliseconds. * p = .05, ** p < .05, *** p < 0.01
